# Identification and validation of copy number variants using SNP genotyping arrays from a large clinical cohort

**DOI:** 10.1186/1471-2164-13-241

**Published:** 2012-06-15

**Authors:** Armand Valsesia, Brian J Stevenson, Dawn Waterworth, Vincent Mooser, Peter Vollenweider, Gérard Waeber, C Victor Jongeneel, Jacques S Beckmann, Zoltán Kutalik, Sven Bergmann

**Affiliations:** 1 Department of Medical Genetics, University of Lausanne, Lausanne, Switzerland; 2 Swiss Institute of Bioinformatics, Lausanne, Switzerland; 3 Ludwig Institute for Cancer Research, Lausanne, Switzerland; 4 Medical Genetics/Clinical Pharmacology and Discovery Medicine, GlaxoSmithKline, Philadelphia, Pennsylvania, USA; 5 Department of Medicine, Centre Hospitalier Universitaire Vaudois, Lausanne, Switzerland; 6 Institute for Genomic Biology and National Center for Supercomputing Applications, University of Illinois, Illinois, USA; 7 Service of Medical Genetics, Centre Hospitalier Universitaire Vaudois, Lausanne, Switzerland

## Abstract

**Background:**

Genotypes obtained with commercial SNP arrays have been extensively used in many large case-control or population-based cohorts for SNP-based genome-wide association studies for a multitude of traits. Yet, these genotypes capture only a small fraction of the variance of the studied traits. Genomic structural variants (GSV) such as Copy Number Variation (CNV) may account for part of the missing heritability, but their comprehensive detection requires either next-generation arrays or sequencing. Sophisticated algorithms that infer CNVs by combining the intensities from SNP-probes for the two alleles can already be used to extract a partial view of such GSV from existing data sets.

**Results:**

Here we present several advances to facilitate the latter approach. First, we introduce a novel CNV detection method based on a Gaussian Mixture Model. Second, we propose a new algorithm, PCA merge, for combining copy-number profiles from many individuals into consensus regions. We applied both our new methods as well as existing ones to data from 5612 individuals from the *CoLaus study* who were genotyped on Affymetrix 500K arrays. We developed a number of procedures in order to evaluate the performance of the different methods. This includes comparison with previously published CNVs as well as using a replication sample of 239 individuals, genotyped with Illumina 550K arrays. We also established a new evaluation procedure that employs the fact that related individuals are expected to share their CNVs more frequently than randomly selected individuals. The ability to detect both rare and common CNVs provides a valuable resource that will facilitate association studies exploring potential phenotypic associations with CNVs.

**Conclusion:**

Our new methodologies for CNV detection and their evaluation will help in extracting additional information from the large amount of SNP-genotyping data on various cohorts and use this to explore structural variants and their impact on complex traits.

## Background

Genetic variation in the human genome takes many forms ranging from large chromosome anomalies to single nucleotide polymorphisms (SNPs). Deletion, insertion and duplication events giving rise to copy number variations (CNVs) are found genome-wide in humans [1-8] and other species [9-12]. Genomic variants can impact both somatic and germ-line genetics. The link between CNVs and inherited diseases is now solidly established (e.g. [13-15]), and copy number plasticity is typical of cancer cells [16]. Such genomic variability, which was identified more than a decade ago using array-based comparative hybridization [17,18], was known for much longer from cytogenetic studies or Southern blots. It has been demonstrated that CNVs near oncogenes or tumor suppressor genes can affect gene expression levels or result in the expression of chimeric fusion genes [18,19]. However, the number and positions of rare CNVs in the human genome are still likely to be underestimated and their contribution to common complex diseases such as diabetes or obesity is unclear. Very recent results demonstrate that rare variants can have very high penetrance in the etiology of morbid obesity [20,21].

The CoLaus (Cohorte Lausannoise) study is a population-based survey started in 2003 to study risk factors for hypertension and cardiovascular diseases [22]. 6188 Caucasian individuals (35-75 years old) from the Lausanne area in Switzerland participated in the study. 5612 individuals were genotyped on Affymetrix 500K SNP chips, and a fraction of these were also genotyped on the Illumina 550 K SNP chips [23]. A number of SNP-based genome-wide association studies (GWAS) that employed the CoLaus data have already been reported [24-30]. Although many other large cohorts including thousands of individuals have been genotyped for SNPs [24,25,31], very few have reported CNV maps [32,33].

It is important to emphasize that most SNP arrays used so far in GWAS of clinical cohorts were not designed for CNV (dosage) detection, but only to call the three possible genotypes of SNPs. Nevertheless, by combining the intensities of the two alleles for a given SNP, it is possible to obtain information also on the copy number state of the SNP locus. However, this is challenging for several reasons: First, when analyzing very large datasets (with several thousands of individuals), it is likely that experiments were conducted at different times and/or by different laboratories, which often introduces strong batch effects for the raw intensities. Thus the first challenge in CNV calling is to ensure proper normalization of these raw data. Second, due to the large noise in the SNP probe intensities in these arrays (even after batch effects have been corrected for), the estimates of copy numbers for a given locus (SNP) are not very reliable. Thus more reliable prediction can only be made by integration of intensities from several neighboring loci, a strategy that is employed by many different CNV detection methods [34-40]. However, this approach makes CNV detection difficult (and sometimes completely fails) in regions with low SNP density. To overcome this limitation, the Illumina (1M) and Affymetrix arrays (Affymetrix 5.0 and 6.0) include more SNP markers and non-polymorphic probes to cover CNV-rich regions. These arrays also received considerable attention from the community and now benefit from a variety of freely available and efficient CNV detection methods [41-47]. These methods also make use of the ratio of allelic intensities which can improve CNV prediction [48]. Until very recently [34], little has been done for Affymetrix 500 K arrays, which were analyzed with software such as dChip [49], CNAG [40], GEMCA [38] and CNAT [39]. All but CNAT are restricted to the Windows operating system and thus are inappropriate for the analysis of large cohorts and for distributed computing on UNIX-based clusters. Software initially developed for Illumina arrays [45,47,50] were modified to allow the analysis of Affymetrix arrays (in particular Affymetrix 5.0 and 6.0 arrays). However the performance of these software on Affymetrix 500 k data has not been intensively tested and for some the software implementation is tedious to use. For example, PennCNV [50] is considered as a very efficient software for CNV analysis. However to analyse Affymetrix 500 K data, several pre-processing steps are needed. These steps rely on external applications (the Affymetrix APT tools) which in their current release do not longer support the pre-processing. While supporting dependencies is a very challenging work in any software development project, it makes it difficult to the user to decide which software to use. In addition, whilst there are now several performance benchmarks for the newest array generation [51-53], assessment of the Affymetrix 500K arrays in large cohorts is still needed.

Finally, while some methods take advantage of the signals from a single or a group of SNPs across the population to predict CNV regions for each individual [41,54,55], there are very few methods to merge individual CNV predictions into regions at the population level: Redon et al. [3] merged CNVs based on the extent of their overlap, whereas Itsara *et al.*[32] manually annotated complex regions.

In the current study we followed two main goals: First, we performed an extensive survey of candidate CNVs in the CoLaus study as detected by SNP genotyping microarrays. We provide a large dataset that can serve as a resource for other studies elucidating human structural variants, and for future association studies of CNVs with the clinical phenotypes measured in CoLaus. Second, since the methods for detecting individual CNV profiles and merging those into consensus regions have not yet been well established, we developed new algorithms for CNV calling and merging, and devised novel techniques to evaluate and compare them with existing methods. Specifically, we compared three existing CNV detection methods with our new method (GMM) that uses a Gaussian Mixture Model to estimate the copy number dosage at each SNP of each individual. GMM models the signal intensity at each SNP across the entire population (cohort) which differs from HMM approaches like CNAT [39], CNAG [40], dChip [49], PennCNV [50] and QuantiSNP [47] that model the signal sample by sample along each chromosome. Other GMM implementations have been successfully used in the past for BAC and CGH array analyses [56-58], but all these different methods (GMMs and HMMs) provide a discrete copy number value (e.g. 0, 1, 2, 3 and 4). It was also proposed to integrate in a single statistical model both the CNV classification and the association with binary traits [59]. Their EM algorithm estimates the copy number state probabilities, but only to use them internally for the association testing. Similar to their approach, our GMM implementation produces (continuous) copy number dosage values that account for uncertainty in the prediction (e.g. due to sample contamination or tumor cell heterogeneity). However, our algorithm couples the calling with CNV merging and focuses explicitly on the copy number region (CNR) calls.

Our GMM was successfully applied to both Affymetrix and Illumina arrays; and is not restricted to SNP array analysis (i.e. is applicable to CGH and qPCR analyses). We also developed two merging strategies, which were applied to create a map of CNV regions for each of the four CNV detection methods. We studied how CNVs predicted by the various algorithms coincided with previously reported variants. We also investigated the concordance in predicting CNVs in a subsample of individuals that were additionally genotyped on the Illumina 550K SNP chips. Finally we compared the sensitivity and specificity of the different approaches using related CoLaus individuals which are expected to share more CNVs than unrelated individuals. Based on these criteria, we demonstrate that our new method outperforms two established CNV detection algorithms and has higher sensitivity than a third method.

## Results

### Identification of copy number variants in Colaus

#### Detection

To detect CNVs in CoLaus, we applied four different CNV detection algorithms to the data from 5612 Caucasians generated with Affymetrix 500 K microarrays: two implementations of the Copy Number Analysis Tool (CNAT [39]) that integrate the SNP intensities by summing their raw (*CNAT.total*) or log-transformed (*CNAT.allelic*) values; Circular Binary Segmentation (CBS [36,37]) and our own algorithm based on a Gaussian Mixture Model, to which we refer subsequently as GMM. We restricted our analysis to autosomes allowing us to use a mixture of males and females as the reference panel. Using these four methods, we assigned copy number values to each probe and each CoLaus individual. (The CBS method only returns segments and their mean signal intensity, which we used to identify SNPs within candidate regions for CNVs if the corresponding ratio was below (loss) or above (gain) a certain threshold, see Methods for more details.)

#### Merging

In the second step we attempted to reduce the complexity of these CNV profiles by merging adjacent SNPs that contained highly redundant information into CNV regions. The first method (“simple merge”) joins neighboring SNPs (on a same chromosome) that have identical copy number values across all CoLaus participants (see Additional file 1: Figure S1A for illustration). This simple approach already significantly reduced the number of SNPs (for example, it compresses 490K autosomal SNPs into 8000 regions for *CNAT.total* and into 40K for *CBS*). However, by nature, this simple scheme leaves the boundaries of CNVs fragmented. I.e. If two adjacent SNPs differ in copy number for at least one subject, they will not be merged together (see Additional file 1: Figure S1B). Thus we devised a refined method, which is based on a principal component analysis (PCA) and self-organizing maps (SOMs). The PCA identifies orthogonal components explaining a significant (e.g. 90%) fraction of the variance. Including these components in clustering or multivariate analyses allows us to remove components that are likely driven by noise and to concentrate on those which, individually, explain a significant fraction of the data variability (i.e. 90%). We then used Self-Organizing Maps (SOMs) to cluster SNPs with similar ‘eigen-value profiles’ in CNV regions (see Methods for details, and Additional file 1: Figure S2 for illustration). For convenience, we refer to this approach as the ‘PCA merge’.

#### Post processing

**Figure 1:**
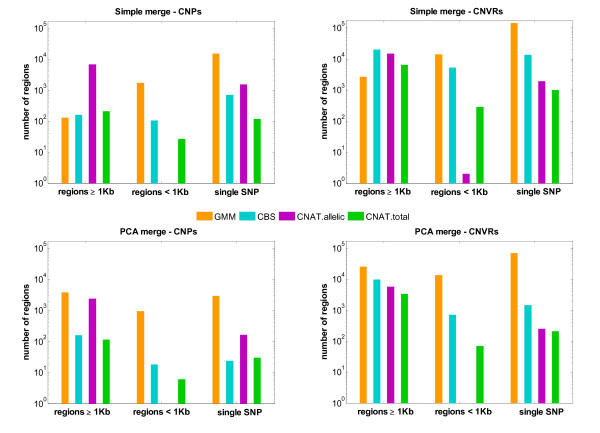
**Counts of CNVs identified with the different methods.** Copy number variants (CNVs) were detected with four different algorithms (see legend) using data generated by Affymetrix 500K SNP arrays for the Cohorte Lausanne (n ≈ 5600). Adjacent SNPs with similar Copy Number profiles were merged into CNV regions using two different approaches: one based on principal component analysis (PCA, bottom panel) and a more simple approach that only merges SNPs with identical profiles (top panel). Copy number polymorphisms (CNPs, i.e. CNVs with population frequency above 1%) are shown on the left. Copy number variant regions (CNVRs, i.e. CNVs with population frequency below 1% but seen for at least five individuals) are shown on the right. In each plot, CNV counts are segregated according to their size.

Next, we excluded any CNV regions found in fewer than five individuals, which roughly correspond to 0.1% frequency. In this study, we considered all remaining CNVs (with frequency >0.1%, refered as ‘all CNVs’) and we also distinguished between Copy Number Polymorphisms (‘CNPs’, CNVs with a frequency greater than 1% in the population) and the remaining ones (i.e. CNVs with population frequency between 0.1% and 1%) to which we refer as Copy Number Variant Regions (‘CNVRs’). The numbers of CNPs and CNVRs predicted by the four different methods and the two merging methods are shown in Figure 1 (see Additional file 1: Figure S3 for detail per chromosome). *CNAT.total* and *CBS* are conservative methods that generate significantly fewer regions than *CNAT.allelic* and *GMM*. The simple merging procedure produces many small regions (<1 kb or single SNPs) which are commonly integrated into fewer larger regions with the PCA-based method (Figure 1, see Additional file 1: Figure S4 for details per chromosome). The PCA-based merging method is able to reduce the total number of regions by 35%, 53%, 67% and 70% for *GMM*, *CNAT.total, CNAT.allelic* and, *CBS*, respectively.

The fraction of the (autosomal) genome effectively covered by these regions is reported in Additional file 1: Table S1 (details per chromosome are provided in Additional file 1: Figure S5). Although *GMM* produces many more CNPs than the other methods, they only cover about 2.4% of the autosomes. *CNAT.allelic* predictions for CNPs cover 12.4% of the autosomes, while *CBS* and *CNAT.total* cover only 1.5% and 0.7% respectively. We also checked the coverage with rare variants (CNVRs), *GMM* had the lowest autosomal coverage of only 9.8%, whereas *CBS* had the highest with 42.4%. CBS predictions for CNPs are rather conservative in the sense that CNPs found with other methods are found for fewer individuals when using CBS (thus much higher genome coverage for CNVRs). Additional file 1: Figure S6 shows the CNV profile on chromosome 1 as predicted by the different methods. This illustrates the dramatic differences between methods and the limited ability of CBS to detect CNPs (despite using optimized thresholds when classifying CBS segments; see Methods for details).

To further investigate at the differences between the four methods, we computed the intersection using CN prediction from 60K independent autosomal SNPs (SNPs that were not in LD in the CEU population, see Supplementary Methods). Only 2.3% of the SNPs composing CNPs were validated with at least three methods (10% with at least two methods) (see Additional file 1: Table S2 and Venn diagrams in Additional file 1: Figure S7). By contrast, 23.5% of the SNPs in CNVRs were found in at least three methods and this number reached 55.3% for at least two methods. Next, we checked pair-wise comparison between the CNV methods (Additional file 1: Table S3). The maximal intersection between two methods is 47% and corresponds to the comparison between all CNVs from *GMM* and *CBS*. Such relatively low overlaps are not uncommon with CNV analysis from SNP genotyping arrays and underline the need for proper replication of any CNV predictions [51,52,60].

In order to evaluate the different detection and merging algorithms, we used three different approaches: (i) A comparison with known CNVs from a public database, (ii) A cross-platform validation using a subset of samples that were also genotyped on the Illumina platform, and (iii) similarity of related individuals with respect to their CNV profiles.

### Comparison with known CNVs

The Database of Genomic Variants (DGV [1]) is a curated catalogue of structural variation in the human genome. We downloaded its content (release 7) and kept only CNVs discovered from SNP or CGH arrays (BAC and ROMA arrays were excluded). We complemented this dataset with predictions from Itsara *et al*. [32] and predictions from the high resolution CNV project [61]. This dataset of “known” CNVs included 17804 autosomal CNVs, whose size ranged from 1 kb to 3 Mb.

**Figure 2:**
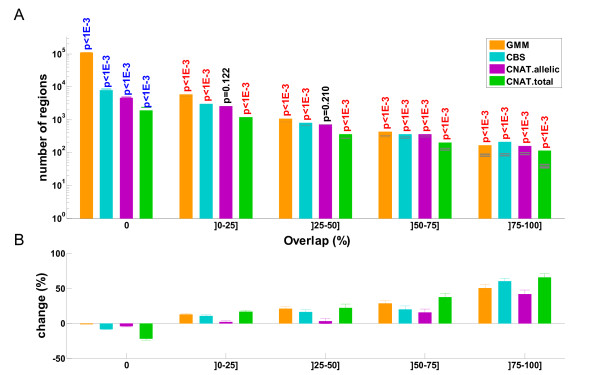
**Overlap between CNVs identified from CoLaus and published CNVs.** A) Counts of CNVs with different methods (see legend) are segregated according to their overlap with CNVs published in the Database of Genomic Variants. Overlap is measured by the Jaccard coefficient, i.e. the ratio between the intersection and the union of two groups of CNVs. Expected counts from (1000 times) reshuffled data are shown in gray (extending over one standard deviation). Estimated p-values are indicated for significant enrichment (red) or depletion (blue), with respect to these controls. Non significant p-values (α > 1%) are shown in black. B) Percentage of changes between observed and expected counts from A. Error bars indicate +/- one standard deviation.

We then computed the overlap between this reference dataset and all CNVs (i.e. CNPs and CNVRs pooled together) generated by each prediction method (Figure 2A). The overlap is reported as the Jaccard coefficient, which is the ratio between the the intersection and the union of two CNVs. A ratio close to one implies that the two CNVs have very similar boundaries; a ratio close to zero indicates a negligible overlap (or no overlap at all if the ratio is equal to zero) and intermediate values correspond to partial overlap (including the case where a small CNV is encompassed by a larger one). Since DGV contains CNVs from much fewer individuals than the CoLaus dataset, it was important to compare the distribution of overlaps with the CNVs generated by the different methods in a controlled setting. Therefore we computed for each method the expected overlap using reshuffled data from 1000 permutations. Estimated *P*-values for observing more or less than expected CNVs with a given overlap are shown in Figure 2A (see Additional file 1: Table S4 for the corresponding *t*-statistics), and the relative excess of observed or expected counts is shown in Figure 2B. We observed that all prediction methods were enriched with respect to the controls for known CNVs (all Jaccard coefficient bins strictly above 50%) and depleted for novel CNVs (Jaccard coefficient of zero). Analyses were also performed for CNPs and CNVRs independently (see Additional file 1: Figure S8). Except for CNAT.allelic, all methods showed significant depletion in novel CNPs. All methods showed significant depletion in novel CNVRs and significant enriched in known CNPs and CNVRs.

### Validation with Illumina arrays

**Figure 3:**
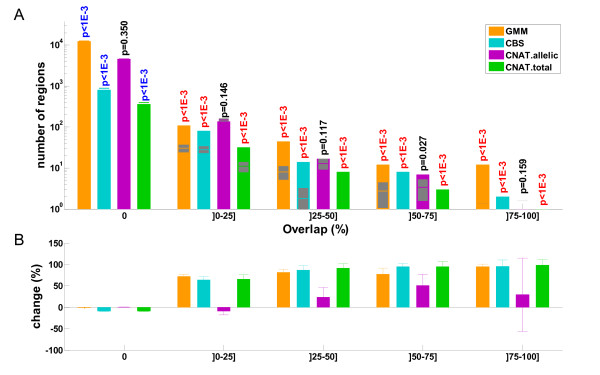
**Overlap between CNVs identified from Affymetrix and Illumina data.** A) Counts of CNVs identified with different methods (see legend) from Affymetrix data are segregated according to their overlap with CNVs identified from Illumina data. The Illumina panel includes a subset of 239 CoLaus individuals. Affymetrix-based CNVs, which did not include at least one individual from the Illumina panel, were excluded from the analysis. Overlap is measured by the Jaccard coefficient, i.e. the ratio between the intersection and the union of two groups of CNVs. Expected counts from (1000 times) reshuffled data are shown in gray (extending over one standard deviation). Estimated p-values are indicated for significant enrichment (red) or depletion (blue), with respect to these controls. Non significant p-values (α > 1%) are shown in black. B) Percentage of changes between observed and expected counts from A. Error bars indicate +/- one standard deviation.

DNA from a subset of 239 CoLaus individuals was assayed on the Illumina SNP platform. In order to obtain a validation set of CNVs, we applied *GMM* and the PCA-based merging algorithm to these data. Note that CNAT is specifically designed for Affymetrix data so it could not be used here. To validate our CNV datasets as predicted from the Affymetrix arrays, we selected those CNVs containing at least one individual that had been assayed on the Illumina arrays. Next, we computed for the overlap between those selected Affymetrix CNVs and the validation CNV collection from the Illumina arrays (Figure 3).

From our overlap analysis, we found that *CNAT.allelic* predictions were not significantly different from random predictions (according to the controls using reshuffled data). This indicates that *CNAT.allelic* is too permissive and that the vast majority of its predictions are likely to be false positives. In contrast, *CNAT.total* had a better specificity than *CNAT.allelic* but identified much fewer CNVs compared to other methods (*CBS* and *GMM*). Both *CBS* and *GMM* performed well (showing depletion of CNVs unique to the Affymetrix data and enrichment of common CNVs). Interestingly, *GMM* predicted many more CNVs than *CBS* and the bias with respect to predictions from reshuffled data was much stronger than for all the other methods (Additional file 1: Table S5). We also performed the above analyses independently for CNPs and CNVRs (both against DGV and the Illumina data, see Additional file 1: Figure S8) and arrived at the same conclusions.

### Predicting relatedness between individuals based on their CNV profile

**Figure 4:**
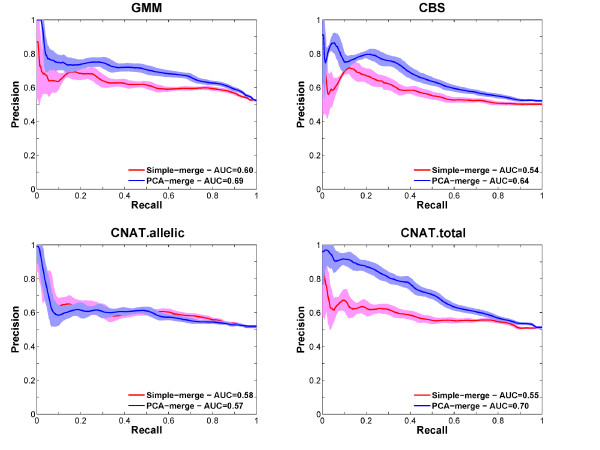
**Performance for predicting relatedness based on CNP profiles generated by different methods.** Each plot shows the Receiver Operator Characteristic (ROC) curve for predicting relatedness between individuals based on the similarity of their CNV profiles generated by different methods (CNV detection algorithms are indicated above each plot and merging procedures by colors). The analysis employed 162 pairs of individuals known to be related and 2000 pairs of unrelated individuals. Curves were made with the mean (solid lines) +/- one standard deviation (light blue or light red surfaces) from 100 permutations. The Precision-Recall Area Under the Curve (AUC) values are shown in the legends.

Pairwise IBS analysis (see Methods and [62]) of the CoLaus genotypes revealed that five individuals had been genotyped twice and the study also included 157 pairs of first-degree relatives (either sibling or parent-offspring relationships). Using this information, we investigated whether predicting the relationship between these individuals would be feasible using exclusively their inferred CNP profiles. To this end we computed the Euclidean distance between the CNV profiles individuals belonging to 162 related pairs and between individuals in 162 randomly selected pairs. Knowing the true relationship status, we computed Precision-Recall (PR) curves for each CNV prediction method and for each merging approach (Figure 4). To evaluate the robustness of these PR curves we reiterated the analysis 100 times with randomly chosen pairs of unrelated individuals.

**Figure 5:**
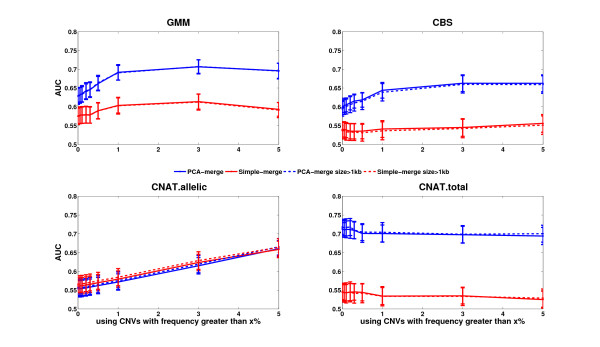
**Performance for predicting relatedness based on CNV profiles generated by different methods.** Each plot shows the Precision-Recall Area Under the Curve (AUC) (Y axis) for predicting relatedness between individuals as a function of CNV frequency (X axis). CNV detection algorithms are indicated on top and merging procedure by colors. Predictions made with all CNV regions irrespective of their length are shown as straight lines and predictions using only CNV regions with length greater than 1 kb are represented with dashed line (both solid and dash lines overlap each other). Curves were made with the mean from n = 100 permutations, +/- one standard deviation around the mean is shown by the thickness of the square points. The analysis employed 162 pairs of individuals known to be related and 162 pairs of unrelated individuals.

All methods had significant prediction power with Precision-Recall Area Under the Curve (PR-AUC) values >0.5. Only the relaxed CNV detection method *CNAT.allelic* did not show a significant difference between the PCA-based and the simple merging approach (both methods had a PR-AUC = 0.58). Interestingly, for all other methods, there was a clear performance advantage of the PCA-based over the simple merging method. Also, these three CNV detection methods, post-processed with the PCA merge, performed better than *CNAT.allelic*. Interestingly, *GMM* and *CNAT.total* had the best PR-AUCs (close to 0.70, see Figure 4). Rank sum analyses did not identify significant difference between GMM and CNAT.total PR-AUCs. (Additional file 1: Figures S9 and S10). We checked whether changing the CNV frequency filter and excluding small regions (<1 kb) would improve the performance (Figure 5). For all methods, there was no significant difference when excluding or keeping such small regions. For *CNAT.allelic*, there was some small improvement when increasing the filter on the CNV frequency, whereas there was no significant change for *CNAT.total* and *CBS*. Apparently, the rather few CNV predictions by *CNAT.total* are of good quality for predicting relatedness as reflected by the high PR-AUCs (0.7). Indeed, *GMM*, which is less conservative, profits from using a filter on CNV frequency significantly, improving its AUC. This improvement is particularly strong in combination with the PCA merge.

## Discussion

In this work, we analyzed CNPs and rare CNV regions within the CoLaus population using four different copy number detection methods and applying two different merging procedures. We also devised various validation strategies to compare the performance of these methods.

### Properties of the PCA merging technique

The simple merging approach is able to concatenate about half a million SNPs into a few thousands regions. Yet, this naïve technique requires discrete copy number predictions and leaves CNV edges fragmented into regions of few or even single SNPs. Therefore we developed a novel merging method, based on a PCA and SOM which, provides a strong improvement over the simple approach as it significantly reduces the number of single SNPs by re-attributing them to larger regions. Also, small regions (<1 kb) were extended either by incorporating single SNPs or by merging them with other small regions.

This new method provides a powerful alternative to the so-called “merge by overlap” method (MbO), commonly used in CNV studies. An inherent limitation of the MbO method is when the underlying CNV is predicted as two distinct regions (i.e. when the predicted CNV locus is disrupted by few probes). Also the MbO requires to discretize CNV predictions (i.e. to convert any region with CN < 2 as a deletion and any region with CN > 2 as a duplication), which results in a significant loss of information (especially in cancer studies where homozygous deletion and focal amplification often play a critical role in the tumorigenesis). Our PCA-merging method allows 1) reconciliation of ‘disrupted’ CNVs, 2) to consider the predicted copy number value without loss of information due to subsequent discretization (i.e. use of continuous copy number prediction) and 3) to ignore (outlier) variation likely induced by noise in the measurement. Our PCA-merge can thus be useful to process the copy number dosage data matrix (of dimension #subjects by #SNPs) and obtain a smaller matrix (#subjects by #CNV regions) for subsequent association studies with a given clinical trait.

### Comparison of the different CNV prediction methods

We demonstrated that *CNAT.allelic* predicts a large number of CNVs. Yet only a relatively small fraction of these could be replicated, indicating that most of the predicted CNVs are likely to be false positives. This is also supported by the fact that CNV profiles generated by *CNAT.allelic* performed worse in predicting kinship. In contrast, *CNAT.total* appears to be overly conservative and is likely to miss subtle, but real CNV events. HMMs are very popular for CNV analysis but our findings underline the difficulty of using parameters that are applicable to different datasets. Ideally, the HMM parameters would need re-evaluation with each novel dataset, which can become tedious in the absence of a ground truth. An obvious improvement of CNAT would include refining HMM transition parameters with Bayesian methods and to co-analyse multiple samples thus improving parameter estimation by combining data across individuals. In addition, summing allelic intensities in the log space (as in *CNAT.allelic*) is adding considerable noise to the CN ratios and thus should be avoided.

Based on our comparative analyses we find that CBS is a robust segmentation algorithm, confirming reports by several independent studies [35,63,64]. Although our *GMM* method, does not explicitly account for probe auto-correlation or allelic intensity ratios, it performs much better than the two CNAT implementations: it recalls more Illumina CNVs (CNPs and rare CNVs) while being depleted in ‘novel CNVs’ with respect to the shuffled controls. GMM and CNAT.total also perform equally well at predicting relatedness between individuals. In addition, *GMM* does not need pre-estimated parameters; the mean and variance of each mixture component (i.e. CN class) are updated from the data using constrained nonlinear optimization [65]. Finally, we observed that our model was able to detect many more CNPs than CBS, suggesting higher sensitivity.

Currently our model only considers deletion, copy neutral, single copy or multiple copies. Since very few homozygous deletions were observed with other applied algorithms, we did not use such a dedicated component in our analysis. Nevertheless, our *GMM* implementation allows for such an extension.

### Validation of CNVs in a large clinical cohort

Validation is an essential part of any CNV discovery project. PCR, Southern blot and many other targeted techniques are useful to predict accurately the copy number at a given locus, but low throughput is a severe limitation when large numbers of CNVs need to be validated. The Database of Genomic Variants is a valuable resource and is useful to compare the ‘known’ (published) CNVs that can be recalled in a large cohort using different methods. However due to the high heterogeneity between studies (e.g. different populations, methods and platforms, unknown false positive rates etc.) and to the absence of medical ascertainment of the subjects, DGV cannot be used to ‘validate’ CNVs (in discovery studies) and must not be used to assess the clinical impact of a given CNV. Instead, for large-scale CNV discovery studies, replicating a number of individuals (e.g. a few hundred) on an independent array platform is a viable option. With the recent reduction in the cost of microarrays, such large-scale replication now becomes affordable. In the context of CNV association with clinical traits, further validations are necessary and would include replication of the association signal in independent cohort(s) (with appropriate clinical ascertainment) as well as CNV validation (for e.g. with MPLA or PCR approaches) in probands. As a complement to replication experiments, one can take advantage of the relatedness between individuals. Deciphering relatedness (if not already known) can easily be achieved by applying simple Method-of-Moments approaches [66-68] to the SNP genotypes. We show that assessing how well the relatedness can be predicted based on the CNV profiles is a powerful technique to gauge the quality of a CNV calling and merging method.

## Conclusions

The combination of our *GMM* and PCA merging algorithms is a useful tool to identify CNVs. They have been successfully applied to a large clinical cohort. The techniques involved here are not limited to data from SNP arrays, they require as input only a matrix of hybridization ratios (for the former) or copy number values (for the latter). Thus they can be applied to data from other platforms such as CGH arrays. Although GMM-like approaches are simplified versions of HMMs, these are simpler to optimize (as opposed to apply pre-trained HMM parameters on a new dataset) and remain powerful tools for the analysis of both large cohort (e.g. CoLaus) and complex dataset, as we recently demonstrated with melanoma [56].

Despite significant improvements in CNV detection and analysis when using the most recent SNP arrays (e.g. new generation Affymetrix arrays [41,54]), there are still many large medical cohorts where SNP data have been collected but CNV analysis has not been reported. This concerns both complex diseases (e.g. [28,69-71]) and cancer (e.g. [72-74]). Hundreds of thousands of individuals have already been genotyped on 500K Affymetrix or 550K Illumina SNP chips, but the corresponding data have not been used for CNV analysis, simply because it is a much more challenging task due to the lack of well-established algorithms and protocols. We hope that the present work will make it easier for researchers to make better use of their data for CNV calling.

GWAS have demonstrated that the genetic variance cannot fully be attributed to SNPs. For example, for highly heritable traits such as height (with 13665 individuals), SNPs only explain 10% of the variance [30]. It has also been shown that, for common traits, the large fraction of heritability cannot be accounted for by CNPs [75]. Thus the identification of rare CNVs with stronger clinical impact, as we recently demonstrated for obesity [20,21], can open up new avenues to explore. Meta-analysis of existing cohorts for CNVs gives more power to detect rare CNVs because unique CNVs in a single cohort can then be supported by different cohorts. But such meta-analyses cannot be used to identify small variants due to the poor SNP density. In such cases, individuals with rare variants should be investigated further with higher density arrays or with genomic sequencing.

With the recent cost reduction in next generation sequencing (NGS), full-genome and exome sequencing become possible even for large cohorts (a few hundred subjects). Already data from several large studies can be retrieved [76-80] and many different algorithms have been developed to mine indels and CNVs [81-87]. Although our GMM method might be applied to predict copy number from sequencing read-depth, it was not developed to this aim. The current Matlab implementation may not be optimal (i.e. not fast enough) and the Gaussian modeling may not be the best option for such analysis (detection methods based on Poisson distribution [81] would be more appropriate). Nevertheless, our PCA merge could be useful for NGS data analyses. These analyses generate massive amount of variants, among which there can be a high number of false positives. Also the predicted variants differ greatly in size (from small indels to larger CNVs) and their boundaries (start and end positions) change between subjects. To some extent, this is similar to the different challenges that occurred in our CoLaus analyses. Therefore our PCA-merge method that is designed to identify consensus CNV regions in large and complex dataset could be of use in the post-processing of NGS structural variants.

## Methods

The implementation of the Gaussian Mixture Model is publicly available at http://www2.unil.ch/cbg/index.php?title=GMM. The algorithm has been implemented in Matlab, both the source code and a compiled version for UNIX 64-bit operating systems are available. The PCA-merging algorithm has also been written in Matlab and the source code is available at http://www2.unil.ch/cbg/index.php?title=PCAmerge.

The source code of the PCA-merging algorithm requires the Matlab Neural Network toolbox, whereas the GMM source code requires the Optimization Toolbox (the compiled GMM version does not have any prerequisites and can be run as a standalone). Both methods require the Statistical Toolbox.

PCAmerge results can be retrieved from http://www2.unil.ch/cbg/index.php?title=File:Colaus_PCAmerge_results.zip.

## Ethics statement

The CoLaus study was approved by the institutional review boards of the University of Lausanne, and written consent was obtained from all participants.

## Samples

The CoLaus design has been previously described [88]. Nuclear DNA was extracted from whole blood and genotyping was performed using Affymetrix 500K SNP chips. Genotype experiments were performed by Affymetrix, Santa Clara CA, following their standard protocol.

## CNV calling

### Copy number analysis tool

We used the Affymetrix GeneChip Genotyping Analysis Software (GTYPE, [23]) to extract, normalize and summarize intensities for both alleles of each SNP. We normalized our data using a sketch-quantile distribution of 50k PM Probes and summarized the intensities using the plier method in RMA mode. (Detailed information can be found in the GTYPE manual.) We first normalized the CoLaus samples versus 30 unrelated CEU Hapmap [89] individuals. Then we used the Affymetrix Copy Number Analysis Tool (CNAT [39]) to attribute a copy number (CN) state to each SNP of all CoLaus individuals with the following encoding: 0 for homozygous deletion, 1 for hemizygous deletion, 2 for copy neutral, 3 for simple gain and 4 for multiple gains. It should be noted that such discrete copy number classification is relative to the median CN in the references. CNAT performs additional normalizations such as PCR bias correction; inter-array normalization when combining NSP and STY arrays; a (100 kb) smoothing function to increase the signal-to-noise ratio; and combines allelic intensities into a CN ratio (CNR). CNAT has two HMM implementations (*CNAT.total* and *CNAT.allelic*), which mainly differ by the way they compute the CN ratios (equation 1 and 2).

(1) $$CNR(CNAT.total)=\log_{2}\left(\frac{S_{A}+S_{B}}{R_{A}+R_{B}}\right)$$

(2) $$CNR(CNAT.allelic)=\log_{2}\left(\frac{S_{A}}{R_{A}}\right)+\log_{2}\left(\frac{S_{B}}{R_{B}}\right)$$

In the above equations, *S* refers to the intensity of the test sample (of an individual) and *R* to the (mean) intensity of the reference panel; A and B refer to the SNP alleles.

The *CNAT.allelic* approach uses the sum of the logs of the allelic signals and is more sensitive to subtle allelic CN changes than *CNAT.total*.

Through QC analyses, we discovered an important batch effect related to the fact that these samples were processed by four distinct centers (respectively with 615, 1666, 1618 and 1736 samples). These batches differed in variance, as revealed with a PCA analysis (Additional file 1: Figure S11). Therefore we normalized data from each genotyping center independently and tested the improvement as a function of the number of references used (see Supplementary Data and Additional file 1: Figure S12). Although Affymetrix suggests that 25 samples are enough for normalization (see CNAT manual www.affymetrix.com), we established that in the presence of strong experimental biases, using many more references performed significantly better (see Supplementary Methods). Thus we re-applied the two CNAT implementations to ratios normalized within each genotyping center and using 280 references, producing much more reliable results than the initial normalization (with 30 references). PCA analysis of the renormalized ratios did not revealed significant differences neither for the genotyping centers (Additional file 1: Figure S13) nor the array set (NSP, STY) (Additional file 1: Figure S14).

### Aroma normalization

In parallel to the normalizations performed using GTYPE, we normalized the CoLaus data with the Aroma.Affymetrix framework [90]. Normalizations were done independently for datasets from each genotyping center with at least 336 individuals (since the Aroma.Affymetrix requires a lot of I/O operations, which can cause a severe drop of the computational performance on shared-network discs, this number of references was decided for optimal computational performances while keeping this number large enough for batch effects correction (see Supplementary Methods). Normalization steps included Allelic Cross-talk calibration [91,92] to correct for differences between SNP alleles; intensity summarization using Robust Median Average and correction for any PCR amplification bias inherent to the Affymetrix SNP platform. To estimate the CNR for a given sample at a given SNP probe, we computed the log_2_ ratio of the normalized intensity of this probe divided by the median across all the samples from the same batch.

### Circular binary segmentation

Circular Binary Segmentation (CBS) has been described as a state-of-the-art segmentation algorithm [36,37]; it identifies change points using maximal t-statistics and assesses segment significance with permutations. We applied CBS, with its default parameters, on the CNRs as obtained by the Aroma.Affymetrix framework. It should be noted CBS only report segments of probes (with their mean log_2_ ratios) and does not provide classification into gains or losses. To this aim, we investigated the distribution of segments’ log_2_ ratios (Additional file 1: Figure S15). This distribution revealed that segments with log_2_ ratios greater than 0.25 or lower than -0.25 were outliers (i.e. ratios greater than 3^rd^ quartile + 1.5 * interquartile range or lower than 1^st^ quartile - 1.5 * interquartile range). A clustering using a three component Gaussian Mixture Model confirmed such data separation. Thus we decided to classify regions having a mean log_2_ ratio greater than 0.25 as gains (CN = 3) and regions with mean log_2_ ratios lower than -0.25 as losses (CN = 1).

### Gaussian mixture models

Raw copy number ratios were smoothed along physical position using Loess filtering with a 41-probe window size (producing the same resolution ~100 kb than the smoothing done in CNAT). This Loess smoothing enables to correct for spatial autocorrelation artifacts due to GC effects [57]. Next, a four component Gaussian mixture model (one component for each of the following copy number states: deletion, copy-neutral, 1 and 2 additional copies) was fitted to the smoothed copy number ratios with a constraint on the differences between the mixture means. Separation between the mixture components is obtained using the simplex search method from Lagarias et al [65].The means of the mixture components were decided not to be fixed as the population mean may not necessarily be two copies. Then, for each individual we determined the probabilities for each of these copy number states (see Additional file 1: Figure S16). The expected copy number was finally assigned as the weighted sum of individual dosage probabilities; for example a SNP with probabilities: 1% for CN = 1, 9% for CN = 2, 85% for CN = 3 and 5% for CN = 4, would have a CN dosage value equal to 2.94 (1*0.1 + 2*0.9 + 3*0.85 + 4*0.05). Evaluation of the GMM performance, using simulated data, is detailed in the Supplementary Methods (see also Additional file 1: Figures S17 and S18).

### Illumina CNV analysis

A subset of 239 CoLaus individuals was analyzed on Illumina arrays (550K version 1 & 3, 1 M [93]). Only SNPs, from the 550K version 1 and 1 M arrays, that could be remapped to the 550K version 3 array (genome assembly build NCBI 36) were used for the analysis. Intensities were normalized within BeadStudio using 120 Hapmap samples. Then copy number ratios (LRR as exported in the Final Report files) were smoothed using Loess smoothing and copy number estimation was performed using GMM. Subsequently CNV predictions were merged into CNVRs with the PCA approach (see below). CNVRs found in only one sample were excluded.

## CNV merging

### Simple merge

Our raw CN data can be represented as a matrix where each element represents the Copy Number status for all individuals (rows) and all SNPs (columns). The “simple merge procedure” consists of combining adjacent SNPs, from a same chromosome, that share the same CN profile across the whole population (see illustration in Additional file 1: Figure S1). This is equivalent to merging strictly identical SNP columns. I.e. to define a CNV region, all the corresponding SNPs from the same subject must have the same predicted copy number. However different subjects can differ in copy number (profile). To avoid creating CNV regions that would encompass long genomic regions with low SNP density, we applied the requirement that two SNPs in the same CNV region should not be further away than 500Kb from each other. This rule did not apply to regions where all SNPs were copy neutral. To perform such merge with the GMM predictions, we rounded the CN values to the nearest integer.

### PCA merge

The PCA merge is a novel merging algorithm for CNV profiles. It includes four steps: (1) each chromosome is partitioned into CNV regions, whose boundaries are a long stretch of SNPs (e.g. 1 Mb in size) that are in the diploid state for all Colaus subjects. (2) For each of these CNV regions, a principal component analysis is performed by analyzing the regional (clipped) CNV profiles (Additional file 1: Figure S2); (3) We then apply a principal component (PC) decomposition of the expected CNV dosage matrix (of size #individuals by #probes). Only the *m* largest components that explain at least 90% of the total variance are then used to derive a (filtered) matrix of SNP eigenvectors (of size *m* by SNPs) which is subsequently used to cluster together SNPs with similar eigenvector profiles. Clustering is done using Self-Organizing Maps (see Supplementary Methods for details about SOMs); (4) strictly adjacent SNPs within a same SOM cluster are merged into final CNV regions.

## Pairwise IBS analysis

Pairwise identity-by-state (IBS) analysis was performed using Plink ([62]). We used a sliding window of 50 SNPs, sliding along in 5 SNP increments. SNPs with a variance inflation factor (VIF) greater than 2 were pruned from each window.

## Availability

**Gaussian Mixture Model:**http://www2.unil.ch/cbg/index.php?title=GMM

**PCA merge algorithm**: http://www2.unil.ch/cbg/index.php?title=PCAmerge

## Competing interests

Dawn Waterworth and Vincent Mooser are full-time employees at GlaxoSmithKline. The funders had no role in study design, data collection and analysis, decision to publish, or preparation of the manuscript.

## Authors’ contributions

SB, ZK and JSB supervised the study. AV generated and analyzed the data. ZK and AV designed the Gaussian Mixture Model. AV designed the merging procedures. BJS and CVJ contributed biological expertise. DW, GW, PV and VM supervised cohort recruitment and/or carried out genotyping. AV, SB, ZK, JSB, BJS and CVJ wrote and edited the paper. All authors commented on and approved the paper.

## Supplementary Material

Additional file 1 Document containing supplementary methods, Figures S1-S18 and Tables S1-S5. Click here for file
