# Supplementary material for: Identification and validation of copy number variants using SNP genotyping arrays from a large clinical cohort
Source: BMC Genomics. 2012 Jun 15;13:241. doi: 10.1186/1471-2164-13-241 (PMC3464625; doi:10.1186/1471-2164-13-241)
Supplement: Additional file 1 — Document containing supplementary methods, Figures S1-S18 and Tables S1-S5. [file 1471-2164-13-241-S1.doc]

# Supplementary methods

## Normalization scheme to account for experimental biases

Principal Component Analysis applied to the CN status of SNPs across CoLaus individuals revealed four distinct clusters of individuals, which corresponds to the four genotyping centers. To correct this batch effect, we performed normalization within each center and used an increasing number of randomly chosen samples (with equal proportions of males and females).

By running the normalization twice on the same individuals but with 2 independent reference panels; we were able to compute the distance between the same individuals (in the two normalization runs) and to compare it to the distance between random defined a score G as:

$$G=\frac{\left|\mu(d1)-\mu(d2)\right|}{\sqrt{std(d1)^{2}+std(d2)^{2}}}$$

where *d1* is the Euclidean distance between CNV profiles of the same individual, but with respect to two different reference panels for normalization (Supplementary Figure 12). For comparison we also computed *d2*, the Euclidean distance between random pairs of individuals; here µ is the mean and *std* is the standard deviation.

This score G measures how well the distribution of distances between pairs of replicates separate from the distance distribution of unrelated pairs. It can be seen an indicator of goodness for a given normalization and is useful to rank normalizations using different number of references. As the number of references increases, the distance between pairs of replicates should become smaller and the separation between replicates and unrelated samples should increase.

We tested normalizations with 30, 120, 200 and 280 references. As expected, the resulting G scores indicated that the normalization improves significantly with the number of references (Supplementary Figure 12). Using 280 references is significantly better than using only 30 references. Using even more references would not significantly improve the normalization; therefore we decided to use 280 references to keep compute times reasonable.

## Intersection between CNV detection methods

Overlap-based approaches are useful to compare the CNV predictions between two distinct methods. But it becomes tedious when comparing results between more than two methods. A way to simplify this problem is to use a common number of elements to compare between the different methods. The full autosomal dataset was subjected to “LD based pruning”, as implemented in plink (Purcell S et al. 2007). We used a sliding window of 100 SNPs, sliding along in 20 SNP increments. SNPs with a variance inflation factor (VIF) greater than 1.2 were pruned from each window. This procedure identified around 60’000 autosomal SNPs. Then, for each method, we created a binary SNP vector, indicating which SNPs where found variant (‘non-copy neutral’) or copy neutral in the CoLaus population. This facilitates the comparison and the number of SNPs found variant in one or more methods can easily be computed. We used such approach both for pair-wise comparison (reporting the Jaccard index between non-copy neutral SNPs) and for generating 4-way Venn diagrams (reporting the number of concordant non-copy neutral predictions between methods). Comparisons were made using 1) all 60’000 SNPs, 2) using SNPs with CNV frequency greater than 1% (referred as ‘CNPs’) and 3) using SNPs with CNV frequency less than 1% (‘rare CNVs’).

## Evaluation of the Gaussian Mixture Model using simulated data

To evaluate the true and false positive rates of our Gaussian Mixture Model, we generated an artificial dataset composed of 5600 individuals, in which the true copy number status was defined and different levels of Gaussian noise was added. This approach is similar to other studies (Lai WR et al. 2005, Willenbrock H and Fridlyand J 2005). In more details, our artificial dataset consists of a distribution of log_2_ ratios (n=5600), where a predefined number of individuals should reflect a deletion state (log_2_ ratio = -1); all remaining individuals are copy neutral thus log_2_ ratios = 0. We then added Gaussian noise to the true copy numbers and tested the ability of our model to correctly detect the underlying copy number state. Supplementary Figure 17 illustrates one test where half of the population is expected to have a deletion.

Supplementary figure 18 shows the performance of our model as a function of the number of individuals sharing the CNV. Each colored curve corresponds to a different level of noise in the data (from low : σ=0.1 to high : σ=0.6). This analysis was repeated 50 times for each point of the curves (mean and standard deviation are shown in the figure). The high TPR and very low FPR demonstrate the good performance of our model.

## SNP clustering using Self-Organizing Maps

Self-organizing map (SOM) is a form of artificial neural network, where the network learns from the data and can perform discrete clustering on the same (learning) dataset. Neural networks are made with nodes (neurons) positioned in the space (a map). Such maps are usually hexagonal or rectangular grids, made with neurons that can connect to their neighbors. Connections between neurons are defined by their distance and by their weights computed from the data. The weight defines the neuron position in the map and is adjusted in a learning phase. This re-adjustment further refines the neuron position and following the learning phase, neurons with similar weights define a same cluster. A specificity of SOMs, by contrast to competitive layers, is that not only the weight from the winning neuron (i.e. the one with weight the closest to the data) but the weight from all neurons in a given neighborhood is updated.

In our analysis, the network is a hexagonal grid of 5 by 3 neurons and is initialized with the newsom Matlab function (Neural Network toolbox). The input vectors are defined by the 'SNP' eigen-vectors resulting from the projection on Principal Components that explain up to 90% of the variance. Subsequently to the training phase, clustering of SNPs is achieved by assigning them the nearest neuron of the SOM. SNPs that belong to the same cluster and that are physically adjacent are merged into the same CNVR.

Since performing a PCA analysis genome-wide or chromosome wise is computationally intensive, we define "blocks of copy number variation" as a chromosomal region flanked with two regions, both with a length greater than a user-specified threshold (e.g. 1Mb) and where all SNPs for all individuals were predicted with the diploid state (CN=2). For each of these blocks, we apply our PCA merging procedure and obtain CNVRs made of SNPs that explain the variance in a similar fashion (i.e. similar pattern of eigen-values).

# Supplementary Figures


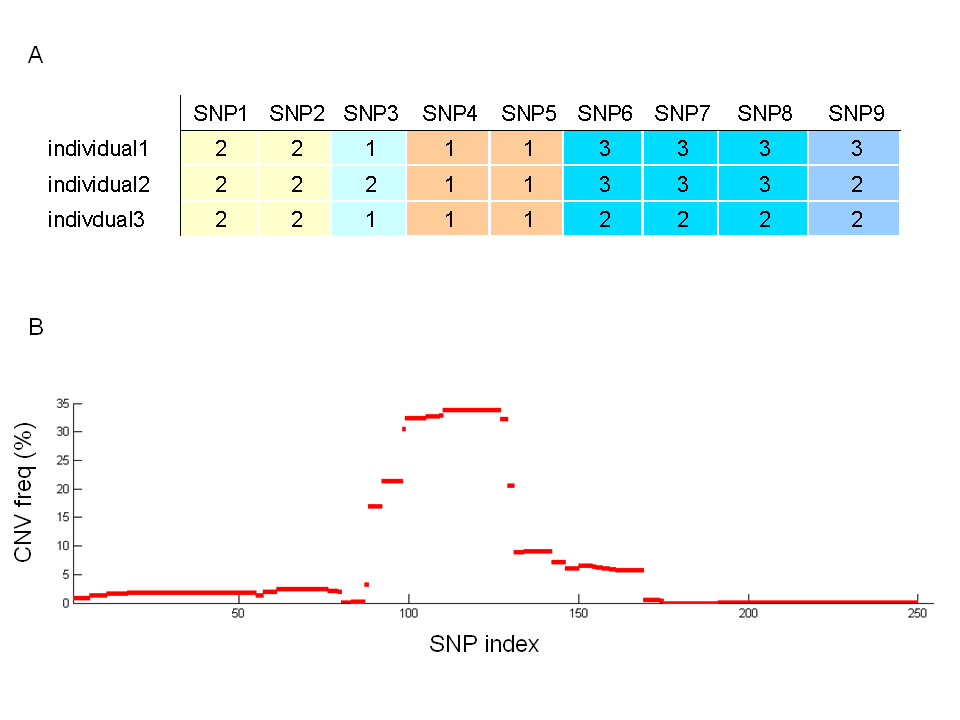


Supplementary Figure 1 Simple merge illustration

**A** Illustration of the simple merge. Data for each chromosome can be represented as a matrix individual (rows) by SNPs (columns). Each matrix element corresponds to the discrete copy number. SNPs are sorted by genomic position. The simple merge consists in merging strictly identical columns into region. In this illustration, the resulting regions would be columns having the same color. **B** Simple merge (red segments) applied on a local SNP window (chromosome3:74.5-76.5Mb) across CoLaus individual. Regions in red regions obtained from simple merge and in blue, regions from the PCA merge. The Y axis represents CNV frequency in the CoLaus population (n≈5600)


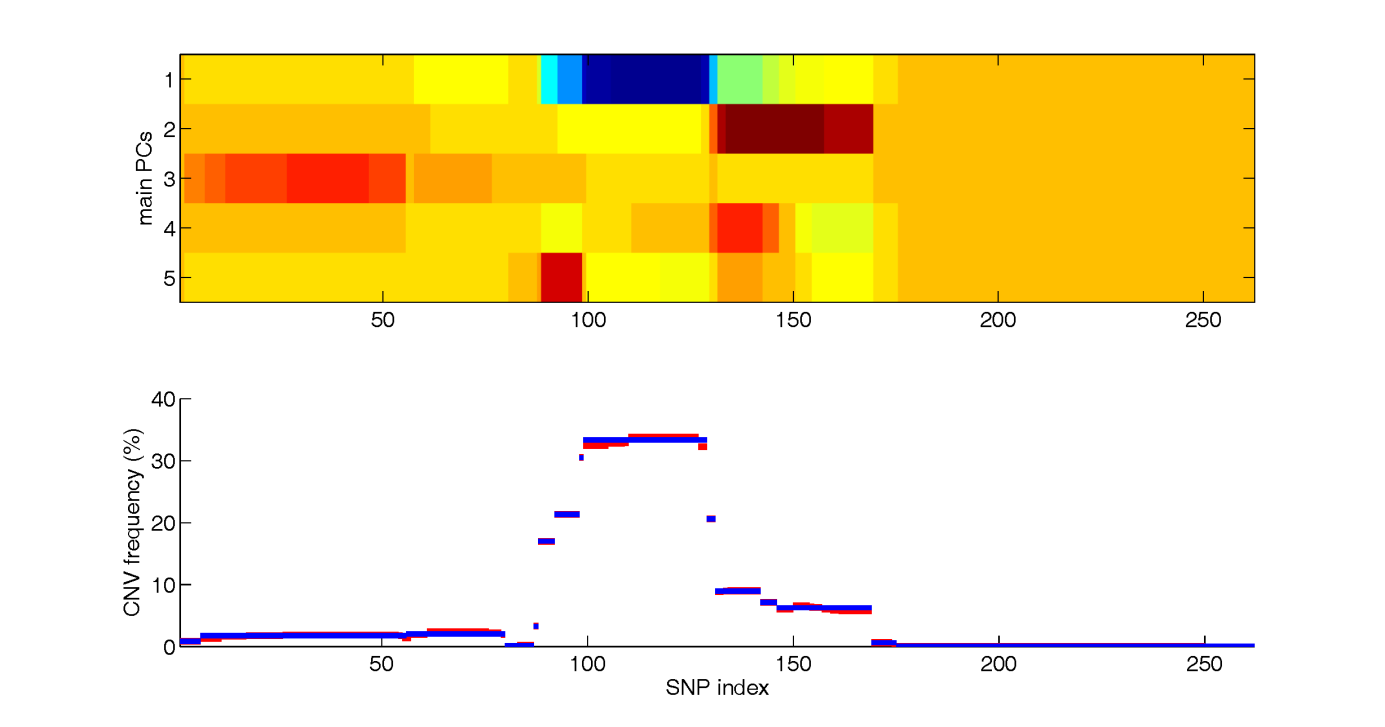


Supplementary Figure 2 Merging SNPs into CNVs using principal component analysis

Top plot shows a principal component analysis (PCA) on a local SNP window (chromosome3:74.5-76.5Mb) across CoLaus individual. The main components are on Y axis and adjacent SNPs are on X axis. The bottom plot shows in red regions obtained from simple merge and in blue, regions from the PCA merge. The Y axis represents CNV frequency in the CoLaus population (n≈5600).


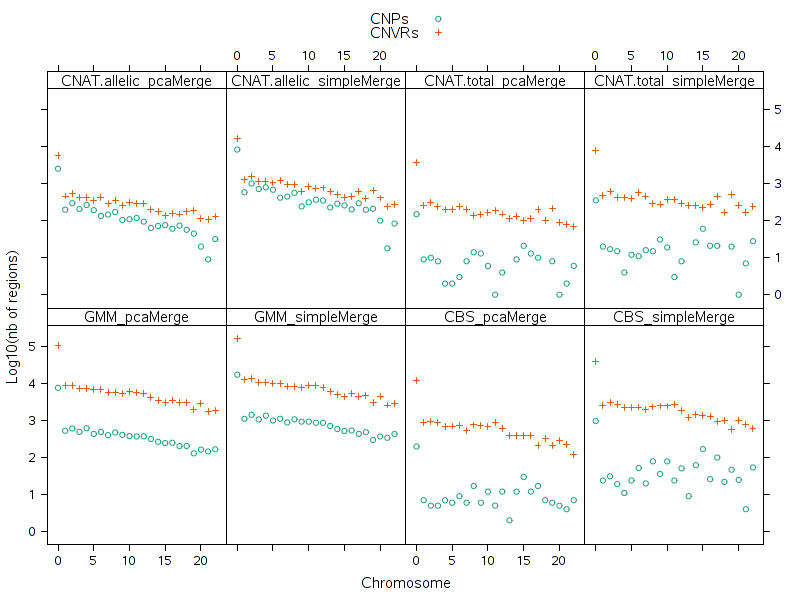


Supplementary Figure 3 Number of CNVs per method

The Y axis indicates the number (log10) of CNPs or CNVRs for each combination of a given CNV detection method method (CBS, CNAT.allelic, CNAT.total or GMM) and merge approach (simpleMerge or pcaMerge). Statistics are provided for each autosome (chromosome 1 to 22) and for all autosomal chromosomes together (chromosome 0).


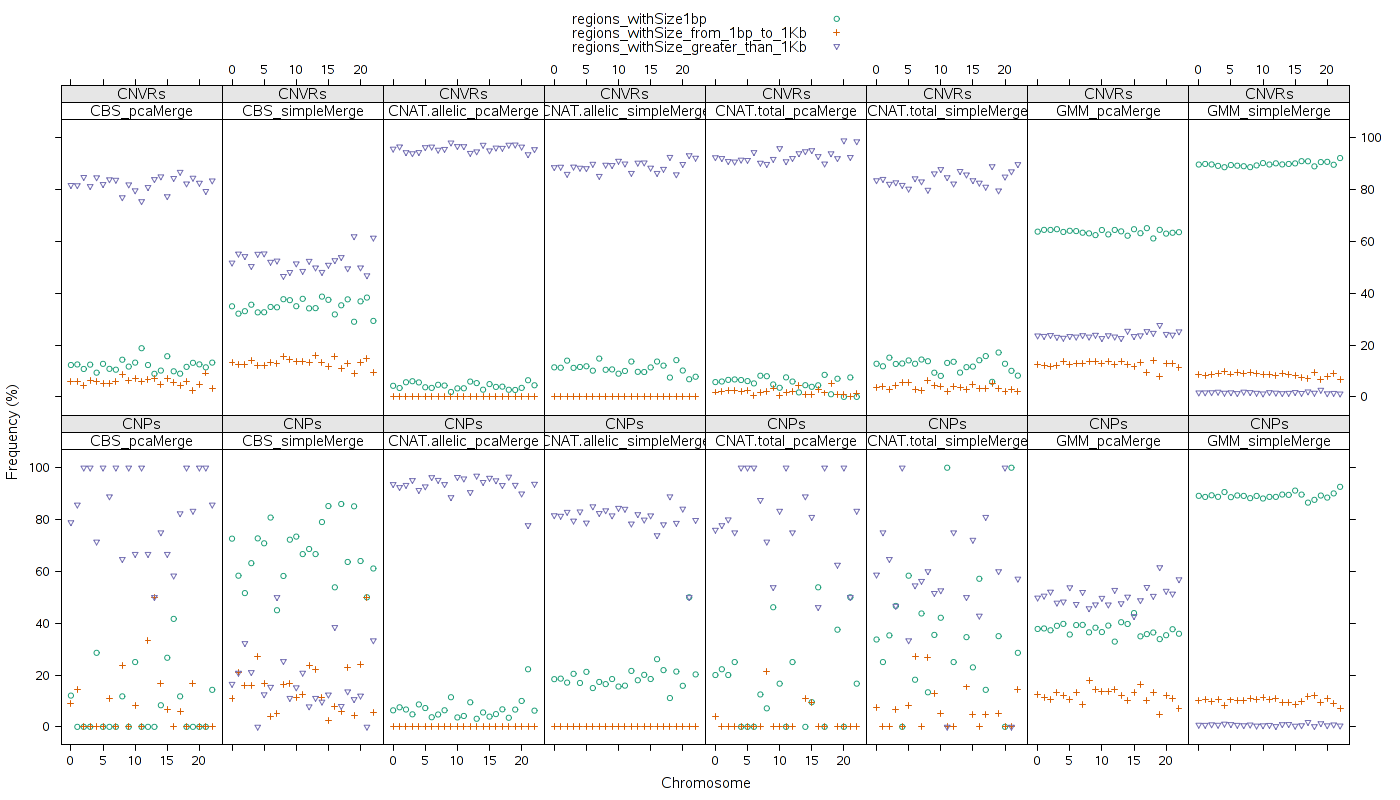


Supplementary Figure 4 Distribution of the CNV length per method and for common (CNPs) and rare (CNVRs) variants

Variants are classified according to their length: single-SNP (1bp), size greater than 1bp and less than 1Kb, size greater or equal than 1Kb. Title from each panel indicates the CNV detection method (CBS, CNAT.allelic, CNAT.total or GMM) and the CNV frequency class (CNPs or CNVRs). Statistics are provided for each autosome (chromosome 1 to 22) and for all autosomal chromosomes together (chromosome 0).


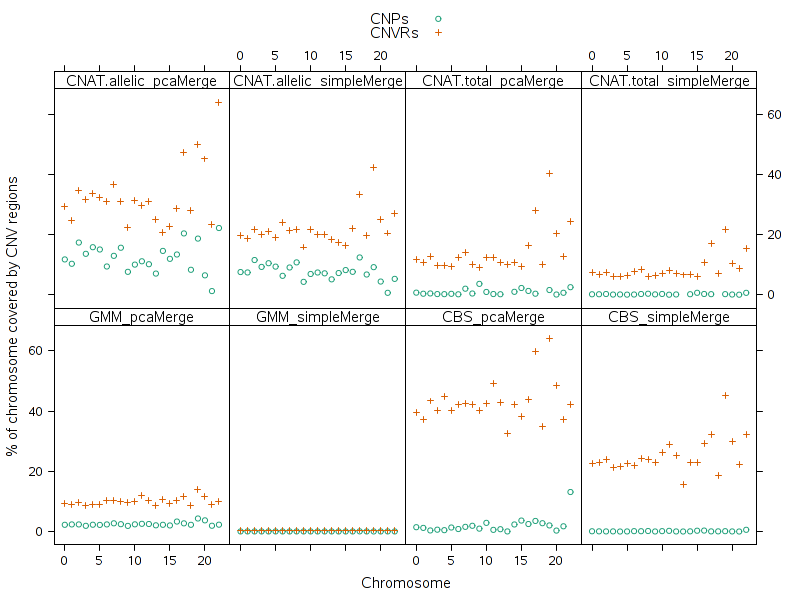


Supplementary Figure 5 Percentage of chromosome covered with common (CNPs) or rare (CNVRs) variants

The Y axis indicates the % of a chromosome that is covered with CNPs or CNVRs for each combination of a given CNV detection method method (CBS, CNAT.allelic, CNAT.total or GMM) and merge approach (simpleMerge or pcaMerge). Statistics are provided for each autosome (chromosome 1 to 22) and for all autosomal chromosomes together (chromosome 0).

| 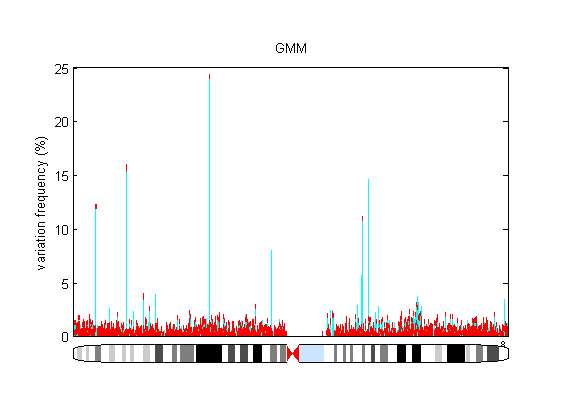 | 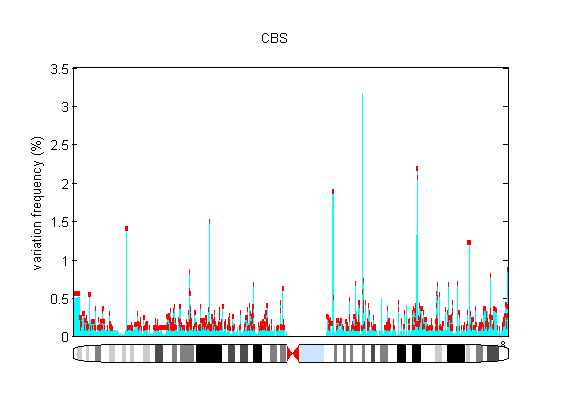 |
| --- | --- |
| 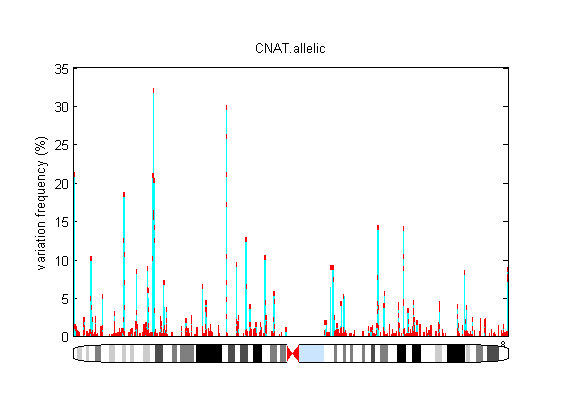 | 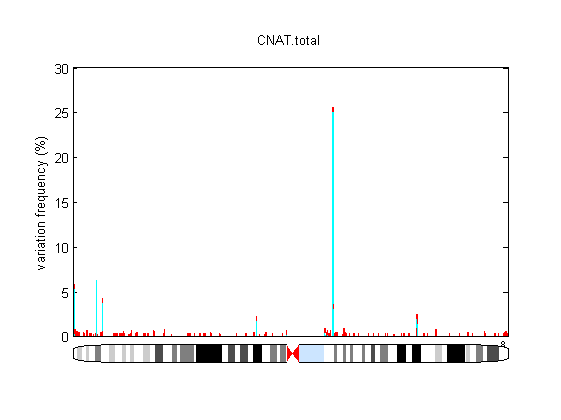 |

Supplementary Figure 6 CNV frequency on chromosome 1

Each plot shows the CNV prediction from a CNV detection algorithm (title indicates the method name). Each cyan bars is a SNP on chromosome 1, the bar height represent the % of CoLaus individuals found as having this SNP as variant (deleted or duplicated). In red are regions defined by the PCA merge, the height shows the maximal CNV frequency found in this region. Only regions with CNV frequency greater than 0.1% are plotted.

|  |  |
| --- | --- |
|  |  |

Supplementary Figure 7 Intersection between CNVs methods

Intersection between the different CNV detection methods using the CNV status at 60k autosomal and independent SNP (i.e. SNPs that are not in LD in the CEU population). The percentage of SNPs found variant in several methods is indicated in Supplementary Table 3.

|  |  |
| --- | --- |
|  |  |

Supplementary Figure 8 Overlap between CNPs/CNVRs and published CNVs/Illumina CNVs

These figures are similar to figures two and three. Each plot title indicates the overlap analysis. Overlap is measured by the Jacquard coefficient, i.e. the ratio between the intersect and the union of two CNVs. Expected counts from reshuffled data (n=1000) are shown in gray (extending over one standard deviation). Estimated p‑values are indicated for significant enrichment (red) or depletion (blue), with respect to these controls. Non significant p‑values (alpha>1%) are shown in black.


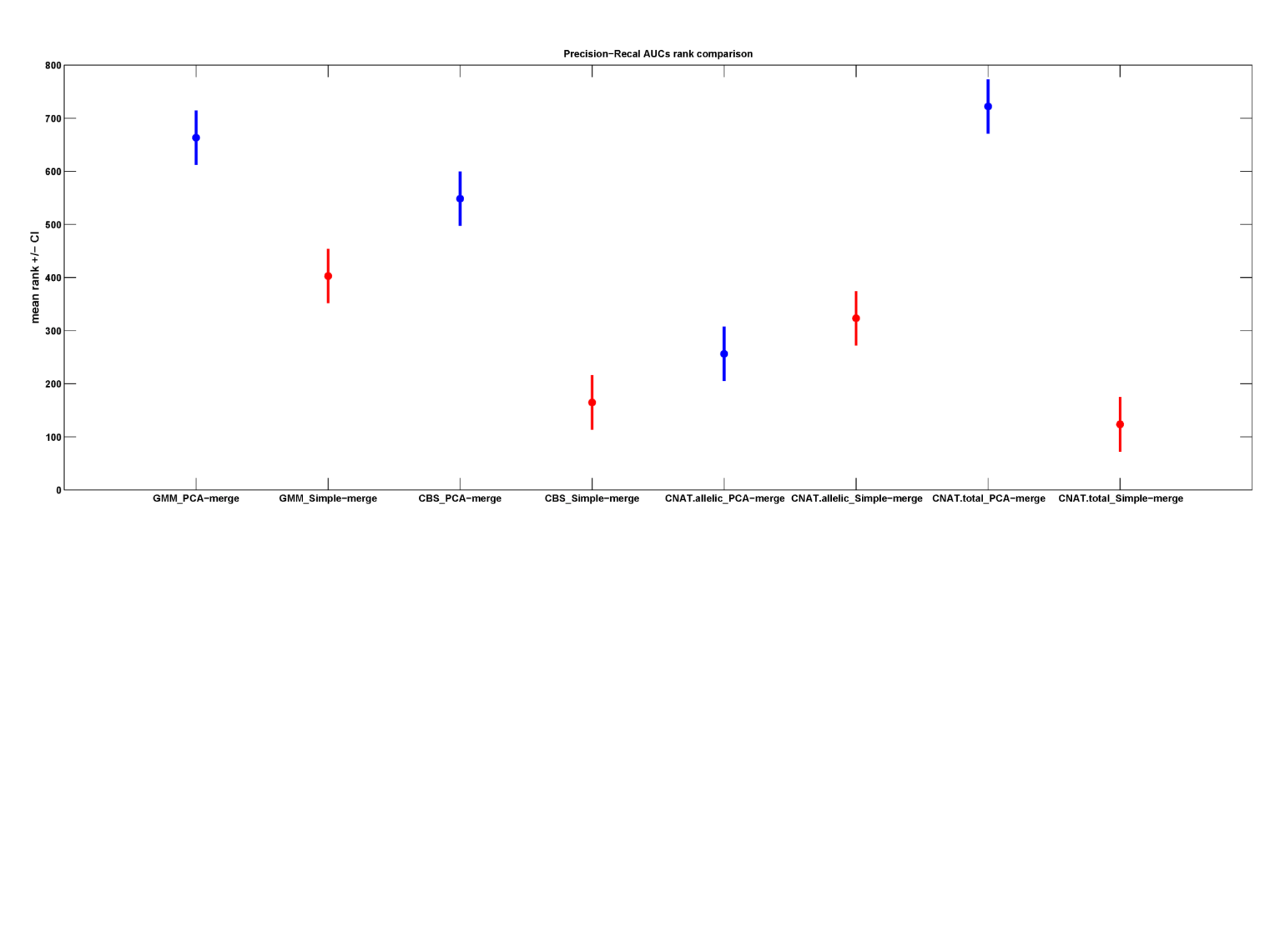


Supplementary Figure 9 Rank comparison of Precision-Recall AUCs when predicting relatedness using all CNPs (frequency >= 1% and no size filter)

Rank comparison (Kruskal–Wallis test) of the performance, from different CNV methods, at predicting relatedness between subjects. Performance was measured as the Precision-Recall AUCs and analyses were re-iterated 100 times with randomly chosen pairs of unrelated individuals. Method names are indicated in the X axis, those used in combination of the ‘PCA merge’ and ‘simple merge’ are shown respectively in blue and red. Dots correspond to the mean group rank and bars indicate the comparison interval. Groups with non-overlapping intervals are significantly different (*P*-values were adjusted for multiple testing issues using a Bonferroni correction, where the number of tests is the number of pairwise comparisons; the resulting adjusted *P*-value was less than 0.05).


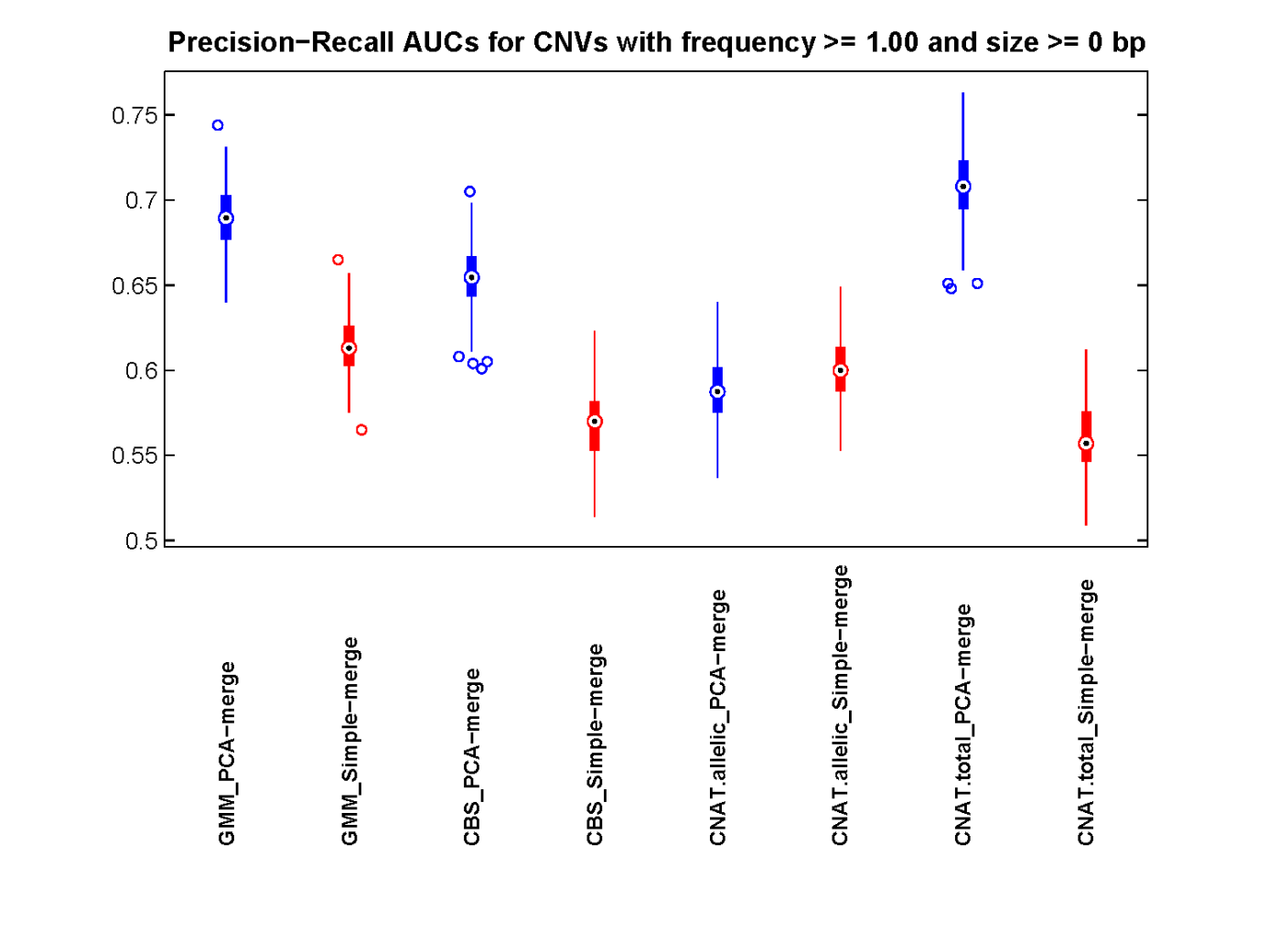


Supplementary Figure 10 Boxplot representation of Precision-Recall AUCs when predicting relatedness using all CNPs (frequency >= 1% and no size filter)

Boxplot representation of the performance from different CNV methods at predicting relatedness between subjects. Performance was measured as the Precision-Recall AUCs and analyses were re-iterated 100 times with randomly chosen pairs of unrelated individuals. Method names are indicated in the X axis, those used in combination of the ‘PCA merge’ and ‘simple merge’ are respectively shown in blue and red.

| 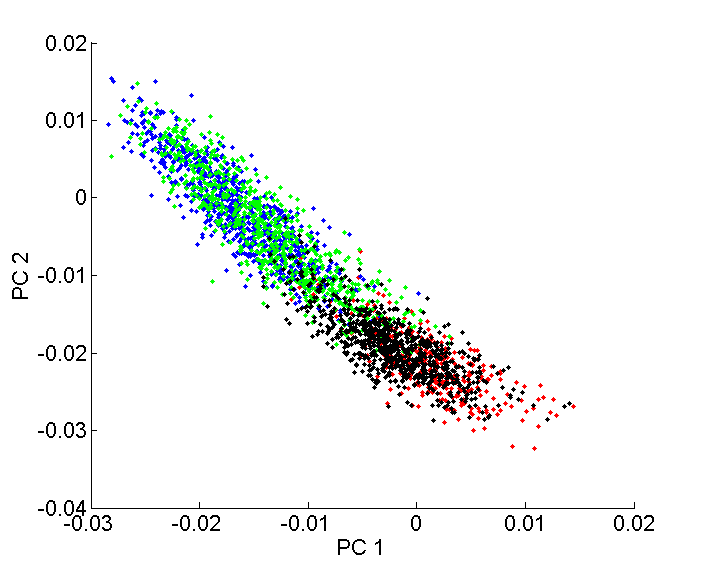 | 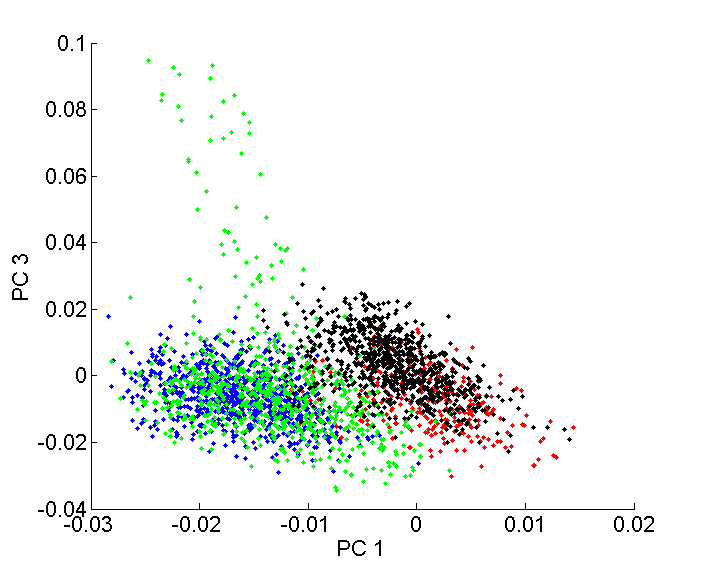 |
| --- | --- |

Supplementary Figure 11 Check for genotyping center bias with the initial data normalization

PCA analysis using copy number ratios normalized using a reference panel of 30 CoLaus individuals. Each dot represent an individual, color indicates in which genotyping center, the sample was processed. Top panel shows the first and second main components, bottom panel shows first and third components.


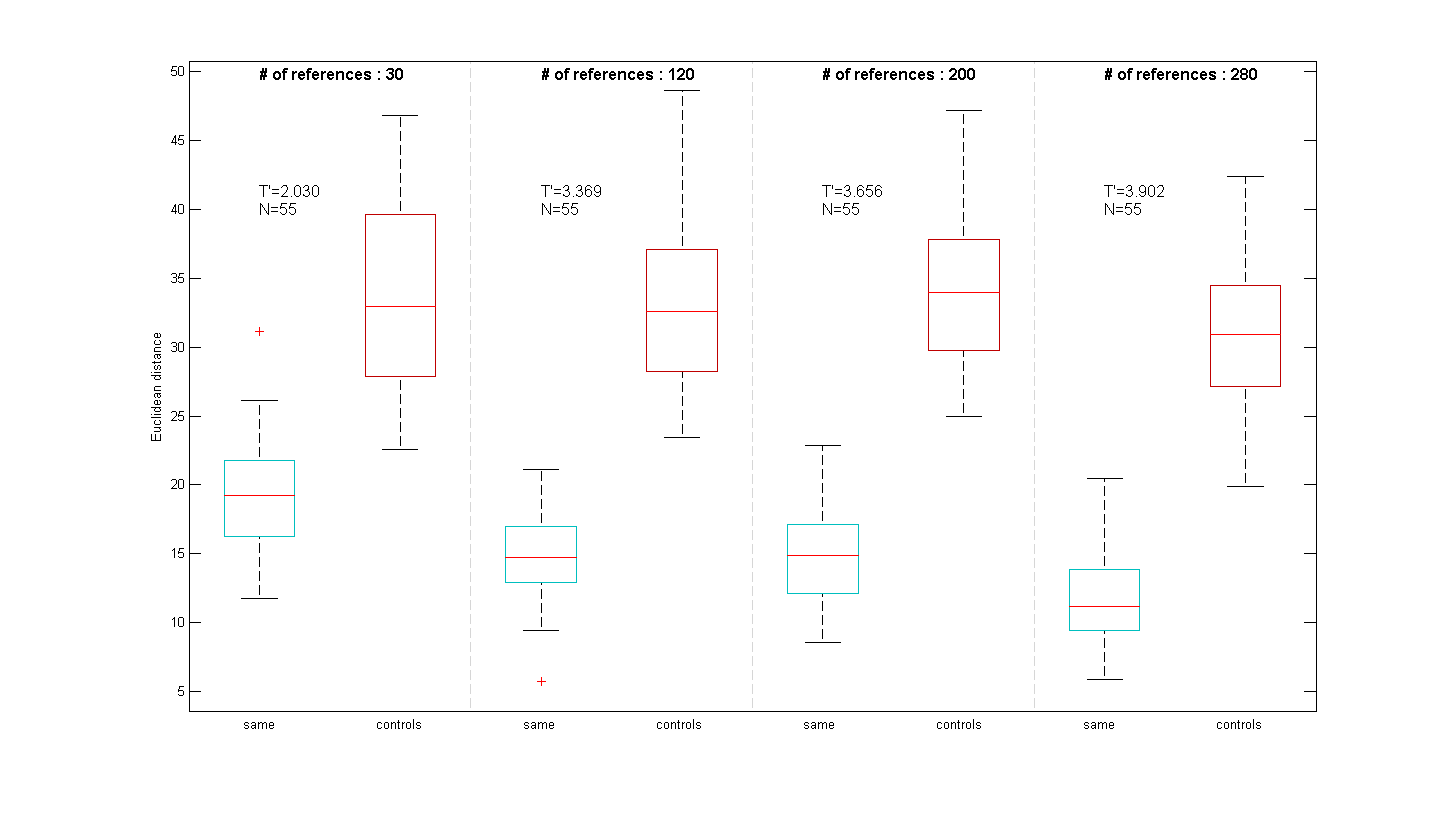


Supplementary Figure 12 Improvement of the normalization as a function of the reference panel size

In cyan are shown the distances (n=55) between the CNV profiles as predicted by two independent reference panel (having the same size) for a same individual. In red are the distances between unrelated individuals predicted by these two reference panels. Different size of reference panel have been tested (30,120,200 and 280). The T’ score is an estimate of the separation between pairs of identical individuals (same) and controls and is computed as:

$$T'=\frac{abs(mean(same)-mean(controls))}{\sqrt{std(same)^{2}+std(controls)^{2}}}$$

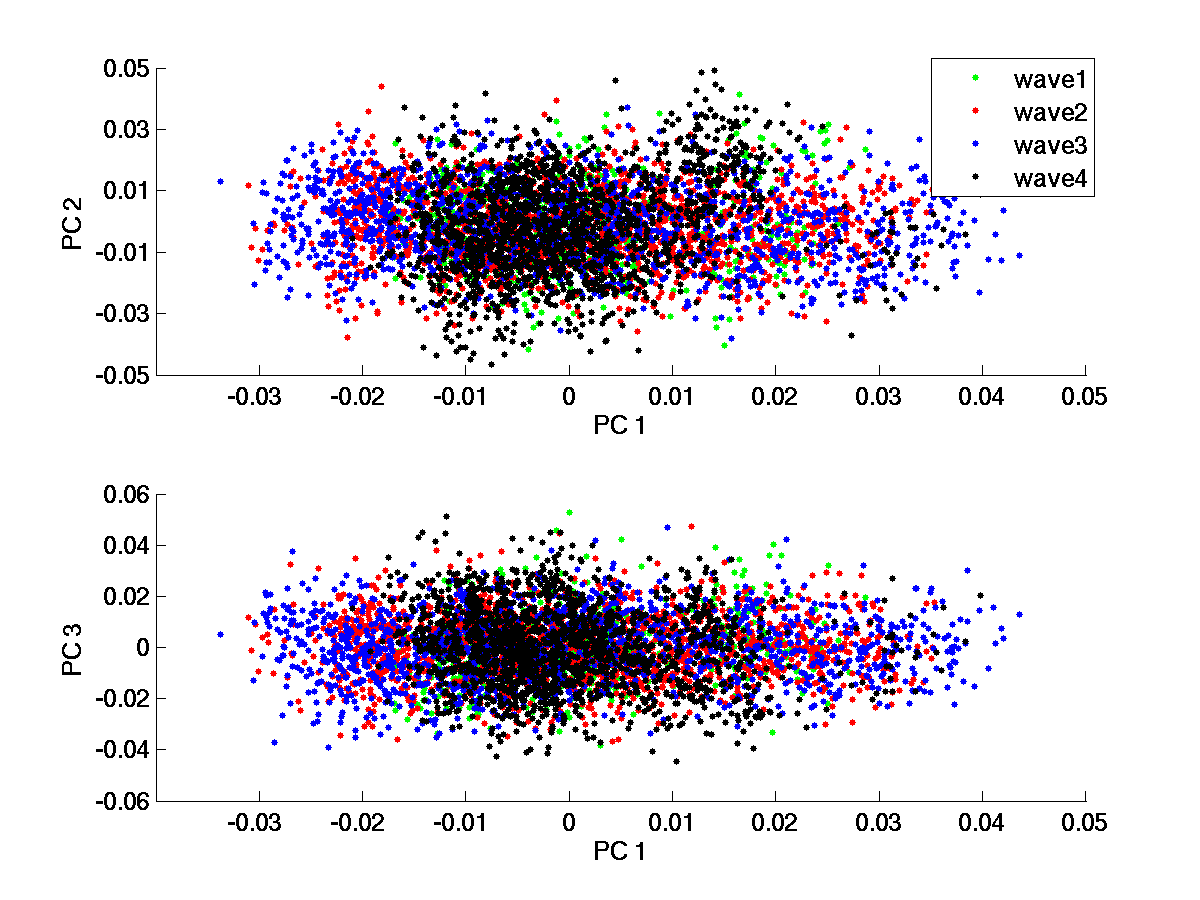


Supplementary Figure 13 Check for genotyping center bias subsequently to data renormalization

PCA analysis using copy number ratios normalized using a reference panel of 280 CoLaus individuals (with samples from both test and reference panel matched for the genotyping center). Each dot represent an individual, color indicates in which genotyping center, the sample was processed. Top panel shows the first and second main components, bottom panel shows first and third components.


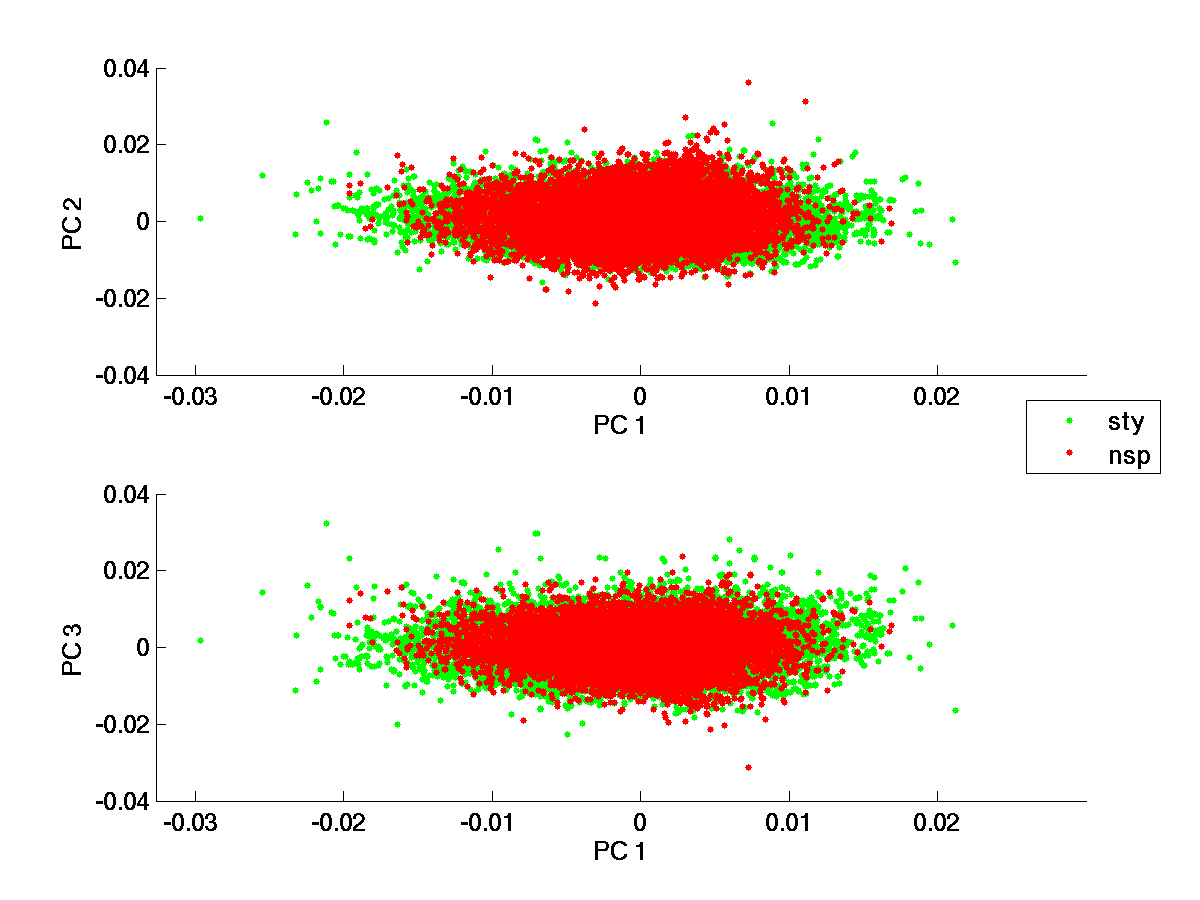


Supplementary Figure 14 Check for SNP array (NSP/STY) bias subsequently to data renormalization

PCA analysis using copy number ratios normalized using a reference panel of 280 CoLaus individuals (with samples from both test and reference panel matched for the genotyping center). Each dot represent a SNP, color indicates on which array the SNP was assayed. Top panel shows the first and second main components, bottom panel shows first and third components.

Supplementary Figure 15 Distribution of CBS segment mean ratios

Distribution of all CBS segments mean log_2_ ratios, detected in the 5612 CoLaus individuals. Left panel shows a boxplot representation. Median is -0.007 (and mean -0.0637), inter-quartile 0.114 and 25^th^ and 75^th^ percentiles are respectively -0.082 and 0.032. Right panel shows an histogram representation, data have been clustered using a 3 components Gaussian Mixture Model (color indicates data points from a same cluster).

Supplementary Figure 16 CNV calling using Gaussian Mixture Model

Top panel, distribution of log2 ratios for all 5613 CoLaus individuals at a single SNP. Bottom panel, matrix of probabilities for CoLaus individuals to have the queried SNP in a deletion state (CN=1), copy neutral (CN=2), with one or additional copy (CN=3 and CN=4). The CN dosage value can be computed as $\sum_{i=1}^{4} P_{i}*C_{i}$ where P_i_ represents the probability at an individual j and a SNP k to be in the copy number state C_i_.

Supplementary Figure 17 Testing the Gaussian Mixture Model on simulated data

**A** Distribution of simulated log_2_ ratios (n=5600) with Gaussian noise (σ=0.2). Half of the individuals have an underlying log_2_ ratio = -1 (deletion) and the remaining log_2_ ratio = 0 (copy neutral) **B** Probabilities, as computed by our Gaussian Mixture Model, for each individual (Y axis) to belong to a given copy number state (X axis) **C** Comparison between the true and predicted copy number state of each individual **D** Contingency table containing the number of individuals correctly or incorrectly predicted.

Supplementary Figure 18 Performance of the Gaussian Mixture Model

Panels show respectively from top to bottom, the True Positive, False Discovery and False Positive rates, when predicting deletions (with predefined frequency, see X axis) in a population of n=5600 individuals. The different curves correspond to simulated data with a given Gaussian noise (see graph legend). Each point is the mean from 50 resamplings, error bars correspond to one standard deviation.

# Supplementary tables

|  | PCA merged | | Simple merge | |
| --- | --- | --- | --- | --- |
|  | CNPs | CNVRs | CNPs | CNVRs |
| GMM | 2.4 | 9.86 | 0.02 | 0.38 |
| CBS | 1.54 | 42.43 | 0.09 | 24.23 |
| CNAT.allelic | 12.4 | 30.88 | 8.08 | 21.19 |
| CNAT.total | 0.73 | 12.71 | 0.15 | 7.79 |

Supplementary Table 1 genome (autosomal chromosomes) coverage of CNVs identified by different methods

CNV detection methods are shown as rows, the merging approach as columns. Distinction is made between CNPs (i.e. CNVs with population frequency above 1%) and CNVRs (i.e. CNVs with population frequency below 1% but seen for at least five individuals). The coverage is expressed as the % of the autosomes.

|  | **CNPs** | **CNVRs** | **All CNVs** |
| --- | --- | --- | --- |
| **% of SNPs found variant in at least 3 methods** | 2.3 | 23.5 | 27.21 |
| **% of SNPs found variant in at least 2 methods** | 10 | 55.3 | 59.17 |

Supplementary Table 2 Percentage of SNPs found copy number variant by several methods

| **CNPS** | **GMM** | **CBS** | **CNAT.allelic** | **CNAT.total** |
| --- | --- | --- | --- | --- |
| **GMM** | 3226 (100.00%) | 317 (8.00%) | 366 (5.58%) | 102 (3.06%) |
| **CBS** |  | 1055 (100.00%) | 228 (5.03%) | 138 (12.22%) |
| **CNAT.allelic** |  |  | 3705 (100.00%) | 172 (4.59%) |
| **CNAT.total** |  |  |  | 212 (100.00%) |
|  |  |  |  |  |
| **CNVRs** | **GMM** | **CBS** | **CNAT.allelic** | **CNAT.total** |
| **GMM** | 25355 (100.00%) | 15720 (39.54%) | 9118 (26.34%) | 5666 (19.48%) |
| **CBS** |  | 30119 (100.00%) | 11543 (31.24%) | 7847 (24.78%) |
| **CNAT.allelic** |  |  | 18376 (100.00%) | 7073 (34.16%) |
| **CNAT.total** |  |  |  | 9401 (100.00%) |
|  |  |  |  |  |
| **ALL CNVs** | **GMM** | **CBS** | **CNAT.allelic** | **CNAT.total** |
| **GMM** | 28581 (100.00%) | 19098 (46.97%) | 12555 (32.95%) | 7155 (23.05%) |
| **CBS** |  | 31174 (100.00%) | 14274 (36.62%) | 8632 (26.84%) |
| **CNAT.allelic** |  |  | 22081 (100.00%) | 8521 (36.77%) |
| **CNAT.total** |  |  |  | 9613 (100.00%) |

Supplementary Table 3 Pairwise comparison between CNV detection methods

Pairwise comparison between the different detection methods using the CNV status at 60k autosomal and independent SNPs (i.e. SNPs that are not in LD in the CEU population). Intersection is reported as the number of SNPs found variant in both methods, the percentage, indicated between the parentheses, corresponds to the intersection divided by the union of SNPs found variant in any of the two methods. The number of variant SNPs in each method (independently of any other methods) is indicated as self-self comparison.

|  | GMM | CBS | CNAT.allelic | CNAT.allelic |
| --- | --- | --- | --- | --- |
| 0 | -12.21 | -10.26 | -3.62 | -10.42 |
| ]0-25] | 8.79 | 6.27 | 1.20 | 6.36 |
| ]25-50] | 7.53 | 4.75 | 0.81 | 4.12 |
| ]50-75] | 7.25 | 4.15 | 3.17 | 6.25 |
| ]75-100] | 9.48 | 13.75 | 7.03 | 11.43 |

Supplementary Table 4 T statistic values for overlap between CNVs identified from CoLaus and published CNVs

The T statistic is computed from the difference between observed overlap and expected counts normalized by the standard deviation of expected counts. Expected counts are inferred from the overlap between reshuffled data (n=1000) and published CNVs. T statistics greater than 2.58 are significant with α=1%. Positive (negative) T statistics indicates enrichment (depletion) with respect to the expected counts.

|  | GMM | CBS | CNAT.allelic | CNAT.allelic |
| --- | --- | --- | --- | --- |
| 0 | -17.68 | -12.67 | 0.31 | -9.69 |
| ]0-25] | 12.14 | 9.78 | -1.01 | 6.81 |
| ]25-50] | 12.81 | 8.45 | 1.06 | 8.59 |
| ]50-75] | 5.49 | 12.28 | 1.95 | 7.78 |
| ]75-100] | 15.14 | 6.13 | 0.34 | 7.18 |

Supplementary Table 5 T statistic values for overlap between CNVs identified from Affymetrix and Illumina data

The T statistic is computed from the difference between observed overlap and expected counts normalized by the standard deviation of expected counts. Expected counts are inferred from the overlap between reshuffled data (n=1000) and CNVs identified on Illumina. T statistics greater than 2.58 are significant with α=1%. Positive (negative) T statistics indicates enrichment (depletion) with respect to the expected counts.
